# Update on Medicinal Plants with Potency on* Mycobacterium ulcerans*

**DOI:** 10.1155/2015/917086

**Published:** 2015-12-08

**Authors:** Patrick Valere Tsouh Fokou, Alexander Kwadwo Nyarko, Regina Appiah-Opong, Lauve Rachel Tchokouaha Yamthe, Mark Ofosuhene, Fabrice Fekam Boyom

**Affiliations:** ^1^Clinical Pathology Department, Noguchi Memorial Institute for Medical Research, College of Health Sciences, University of Ghana, P.O. Box LG 581, Accra, Ghana; ^2^Antimicrobial Agents Unit, Laboratory for Phytobiochemistry and Medicinal Plants Study, Faculty of Science, University of Yaoundé 1, P.O. 812, Yaoundé, Cameroon; ^3^Department of Pharmacology and Toxicology, School of Pharmacy, College of Health Sciences, University of Ghana, P.O. Box LG 43, Legon, Ghana; ^4^Institute of Medical Research and Medicinal Plants Studies (IMPM), P.O. Box 6163, Yaoundé, Cameroon

## Abstract

*Mycobacterium ulcerans* disease has been a serious threat for people living in rural remote areas. Due to poverty or availability of traditional medicine these populations rely on herbal remedies. Currently, data on the anti-*Mycobacterium ulcerans* activity of plants, so far considered community-based knowledge, have been scientifically confirmed, concomitantly with some medicinal plants used to treat infectious diseases in general. Products derived from plants usually responsible for the biological properties may potentially control* Mycobacterium ulcerans* disease; numerous studies have aimed to describe the chemical composition of these plant antimicrobials. Thus, the present work provides the first compilation of medicinal plants that demonstrated inhibitory potential on* Mycobacterium ulcerans*. This work shows that the natural products represent potential alternatives to standard therapies for use as curative medicine for* Mycobacterium ulcerans* disease.

## 1. Background

*Mycobacterium ulcerans* disease has been a serious threat for people living in rural remote areas. Due to cultural belief, convenience or inaccessibility of modern therapy, in many rural folk, usually relies on traditional medicine for ulcer management [1]. Besides, many people with* M. ulcerans* disease suffer mutilation and amputation because modern treatment seems to be expensive and also associated with side effects [2]. These traditional medicines are usually sold in markets and public places or administered by traditional healers in their clinics. Most remedies are mixtures of two or more plants species and solvents used including water, palm wine, or oils. Health problems are often treated through self-medication, first with the popular pharmacopoeia [3, 4] or specialized pharmacopoeia from traditional healers for difficult health problems.

Traditional treatment for* Mycobacterium ulcerans* disease is done in four steps that involve diagnosis, necrosis ablation, wound curing, and exorcism [1]. The use of medicinal plants takes place in the second and the third stages of the treatment process [1]. Although medicinal plants may play a significant role in Buruli ulcer case management and control of the disease at an affordable cost for the local population, no attempt has been made to document potent medicinal plants against Buruli ulcer. We previously compiled ethnopharmacological data on plants used traditionally to treat Buruli ulcer which addressed only the activities of ethnopharmacolocally used medicinal plants excluding plants for which there was no report on their use against Buruli ulcer. In this review we provide a comprehensive list of medicinal plants that show activity against* M. ulcerans*. The list includes plants that are traditionally used in Buruli ulcer treatment.

## 2. An Overview of the Assay Methods Used to Estimate Antimycobacteria Activity

### 2.1. Antimicrobial Assays

Several* M. ulcerans* strains and isolates have been successfully adapted to* in vitro* culture. They were employed in the activity screening described. Two different assays were used to determine the antimicrobial activity of the extracts, fractions, and pure compounds and where possible were compared to commercially available antimicrobial agents, such as rifampicin (MIC = 2 *μ*g/mL) [20].

#### 2.1.1. The Proportion Method

The proportion method, which is still the main method used for drug or products screening of* Mycobacterium ulcerans* by many researchers, applied Löwenstein-Jensen medium. This involved the use of a drug containing media associated with or without dilution of the drug and drug-free media. A standardized inoculum was applied to the drug containing and drug-free media and incubated. After numbering the colonies formed on drug containing medium and on drug-free medium, the MIC was defined as the minimum concentration that inhibited at least 99% mycobacteria [53]. From the studies reviewed, serial dilution of the plant extracts at concentration ranging from 0.4 *μ*g/mL to 50 *μ*g/mL (25% vol/vol to 0.20% vol/vol) [5] or 0.5 to 50 mg/mL [33, 43] was incorporated into Löwenstein-Jensen media. Thereafter, inocula size of 10^4^ to 10^5^ mycobacteria/mL was applied and incubated for 8 to 12 weeks at 30–33°C [5, 33, 43].

#### 2.1.2. The Resazurin Microtiter Assays (REMA)

Resazurin or alamar Blue-based assays are sensitive and powerful tools used to detect growth inhibition of a given substances in various concentration ranges on mammalian cell lines and microorganism including mycobacteria [66]. The resazurin microtiter assay was first proposed as a simple, rapid, and cheap alternative for* Mycobacterium tuberculosis* susceptibility testing by Martin et al. [67] and was then adapted by Yemoa et al. in 2011 [21] for* Mycobacterium ulcerans* susceptibility testing. This method is performed usually in the 96-well microplates and can be read with a spectrophotometer or spectrofluorometer or visually without any equipment. In the latter case, which is the one used in the review studies, the minimum inhibitory concentration (MIC) is defined as the lowest concentration of extract that prevents a color change of resazurin from blue to pink. In fact, viable mycobacteria will reduce the blue resazurin into the pink-coloured resorufin. In all cases, extracts or compounds were serially diluted in a 96-well plate and incubate with the inoculum of mycobacteria 10^5^ cfu/mL in Middlebrook 7H9 broth supplemented with oleic acid-albumin-dextrose-catalase with concentration ranging from 7.81 to 12,500 *μ*g/mL. After 15 days of incubation, resazurin was added and a colour change was visible after additional 48 hours of incubation [11, 21].

### 2.2. Medicinal Species with Anti-*Mycobacterium ulcerans* Activities

Several authors have reported medicinal species with biological activity against* M. ulcerans in vitro*. In this work, we have reviewed 33 species either alone or in combination for their potential on* M. ulcerans in vitro* (Table 1).

#### 2.2.1. *Aloe vera* (L.) Burm. f

*A. vera* is an evergreen perennial medicinal plant known for centuries for its beauty and medicinal and skin care properties [51]. Classically,* A. vera* has been used for its wound healing potential [51]. Recently Seefeld et al. [68] reported its use in the traditional treatment of Buruli ulcer while Addo et al. [5] have demonstrated its* in vitro* activities on 7 different* M. ulcerans* strains and isolates with MIC mean value of 40 *μ*g/mL (Table 1). Phenolic compounds such as chromone, anthrone, anthraquinone [52], aloin, and emodin have been identified and are known for their antibacterials effect [51]. Also, its* anti-inflammatory action through the* inhibition of the cyclooxygenase pathway and reduction of prostaglandin E2 production from arachidonic acid was reported. Recently, the novel anti-inflammatory compound called C-glucosyl chromone was isolated from gel extracts [51] of* A. vera*. The anti-inflammatory activity combined with the antibacterial effect and the wound healing activity of* A. vera* support its use in Buruli ulcer case management. In fact, its high water content can keep the wound moist and increase epithelial cell migration. In addition, its content of glucomannan, a mannose-rich polysaccharide, and gibberellin, a growth hormone, interacts with growth factor receptors on fibroblasts and macrophages, thereby significantly increasing collagen and proteoglycan synthesis, and the degree of collagen cross linking after topical and oral administration resulting in accelerated tissue repair [51, 52]. Furthermore, acemannan, saponin, and genin isolated from* Aloe* leaves have been shown to accelerate wound healing [52, 69, 70].

#### 2.2.2. *Alstonia boonei* De Wild. 

*Alstonia boonei* is a large deciduous evergreen tree, usually up to 45 m tall and 1.2 m in diameter, and is also called devil tree in tropical and subtropical Africa, Central America, and Australia [71]. The plant is used to treat various diseases across Africa and the stem bark has been listed as an agent useful for treatment of ulcer or wounds [72]. Addo et al. [5] reported* in vitro* activity of infusion of leaves on 7* M. ulcerans* strains and isolates with MIC mean value of 40 *μ*g/mL (Table 1). A wide array of chemical compounds, including alkaloids (echitamine, echitamidine, voacangine, akuammidine, N-formylechitamidine, N*α*-formyl-12-methoxyechitamidine), tannins, iridoids (boonein, loganin), steroids, saponins, glycosides, flavonoids, and terpenoids and triterpenoids (lupeol, ursolic acid, and *β*-amyrin) [19, 71–73], have been isolated from the plant that might support its reported* Mycobacterium ulcerans* inhibitory effects.

#### 2.2.3. *Pupalia lappacea* Juss. 

*Pupalia lappacea* is a widespread medicinal plant found in savannah and woodland localities as well as forests in tropical Africa and in Asia [74]. It has been used in the management of wound [5, 6] and its leaves extracts have shown inhibitory activity on 7* M. ulcerans* strains and isolates with MIC mean value of 40 *μ*g/mL (cited as 1 : 5 (20% w/v)) (Table 1) [1]. Chemical investigations show that* P. lappacea* leaf extract contains alkaloids, glycosides, flavonoids, saponins, tannins, coumarins, terpenoids, and steroids such as 1-docosanol, stearic acid, stigmasterol, sitosterol, N-benzoyl-L-phenylalaninol acetate, setosterol-3-O-D-glucopyranoside, stigmasterol-3-O-D-glucopyranoside, and 20-hydroxyl ecdysone. These compounds have been shown to promote the wound healing process in animals and humans. Stigmasterol found in the plant has been shown to exhibit haemostatic and anti-inflammatory activities. Similarly, 20-hydroxyl ecdysyne also present in the plant promotes protein synthesis and wound healing in animals and humans. Since some of the compounds contained in* P. lappacea* leaves extract have antibacterial activities and also promote the wound healing process, the plant extract may exhibit wound healing activities as claimed by the traditional practitioners [6–8].

#### 2.2.4. *Lannea nigritana* (Sc. Elliot) Keay

*Lannea nigritana* is a small tree of 3–6 m of height found in the tropical rain forest [14]. It is used in traditional medicine for the treatment of various infectious diseases [14] including wounds [5]. The leaves of the plant showed activity on 7* M. ulcerans* strains and isolates with MIC mean values of 40 *μ*g/mL. Phytochemical studies on this plant are scanty. Meanwhile the plant is known to contain tannins and phenolic compounds (Lanneanol) [5].

#### 2.2.5. *Aglaonema commutatum* Schott. 

*Aglaonema commutatum* is a common ornamental plant used to treat BU that showed MIC mean value of 40 *μ*g/mL on 7* M. ulcerans* isolates (Table 1) [5]. Besides, solution from washings of leaves showed inhibitory effects on growth of bacteria such as* E. coli*,* P. aeruginosa*, and* S. aureus* [75] which are commonly involved in wound infection. All parts of the plant contain the active constituent calcium oxalate crystals [23].

#### 2.2.6. *Ageratum conyzoides* (L.) L

*Ageratum conyzoides* is an annual herbaceous plant used in African traditional medicine for the treatment of wounds, burns, and ulcer [76]. Addo et al. [5] demonstrated the leaves activity on 7* M. ulcerans* strains and isolates with MIC mean value of 40 *μ*g/mL (Table 1).* A. conyzoides* contains phenolic compounds, methoxylated flavonoids and chromenes, and pyrrolizidine alkaloids, pyrrolizidine alkaloids lycopsamine, dihydrolycopsamine, and acetyl-lycopsamine, and their* N*-oxides [29].

#### 2.2.7. *Cleome viscosa* L

*Cleome viscosa* is commonly known as tickweed, wild mustard, or spider plant that occurs in woodland and grassland. It is a weed found in fallow land, fields, roadsides, and wasteland. It often grows on sandy soils but sometimes grows on calcareous and rocky soils. It is a widely distributed herb with yellow flowers and long slender pods containing seeds [77]. The leaves and whole plant of* C. viscosa* are used as a folk remedy to cure wounds, ulcers, inflammations, and skin infections [27]. Its leaves have shown MIC mean value of 40 *μ*g/mL (cited as 1 : 5 (20% w/v)) on 7* M. ulcerans* isolates (Table 1) [5]. Methanolic extract of the aerial parts of the plant is also reported to have significant wound healing properties on experimentally induced excision and incision wound models in rats in addition to its previously reported analgesic, antimicrobial, and antiulcer activities [77]. All of this supports the traditional use of the plant. Its content of alkaloids, tannins, saponins, flavonoids, terpenes, and carbohydrates [27, 28] may be probably responsible for the observed wound healing activity.

#### 2.2.8. *Phyllanthus fraternus* G. L. Webster

*Phyllanthus fraternus* commonly is a small, erect, annual herb that grows 30–40 cm in height [78] indigenous to the rainforests of the Amazon and other tropical areas throughout the world [79]. The plant has numerous uses by indigenous peoples to treat blennorrhagia, colic, diabetes, dysentery, fever, flu, tumors, jaundice, vaginitis, dyspepsia, and pain [80].* P. fraternus* has previously demonstrated an MIC mean value of 40 *μ*g/mL (cited as 1 : 5 (20% w/v)) on 7* M. ulcerans* (Table 1) [5] isolates and strains. It has been shown to contain alkaloids, steroids, glycosides, tannins, saponin, flavonoids, other compounds (estradiol, corilagin, ellagic acid, gallic acid, rutin, gernanine, rutinoside, lupa, lupeol, and methyl salicylate), and saponins (triacontanal, triacontanol) [45, 79]. The reported antimicrobial activity may be attributed to the presence of some of the reported secondary metabolites. However, the active compound(s) known to give this observed activity need to be identified.

#### 2.2.9. *Bridelia ferruginea* Benth

*Bridelia ferruginea* Benth. is a gnarled shrub which can reach the sizes of a tree in suitable condition [30].* B. ferruginea* bark is used for treatment of bacterial infections on wound [81] and has been shown to inhibit the growth of 7* M. ulcerans* strains and isolates with an MIC mean value of 40 *μ*g/mL (cited as 1 : 5 (20% w/v)) (Table 1) [5]. The presence of phytochemical such as polyphenols, steroids, saponins, tannins, terpenoids, and alkaloids [30, 31] may support the demonstrated antimicrobial activity against mycobacteria.

#### 2.2.10. *Senna occidentalis* (L.) Link

*Senna occidentalis* (syn.* Cassia occidentalis*) is an annual or perennial plant [39] that has been used as natural medicine in rainforests and tropical regions as laxative, analgesic, febrifuge, diuretic, hepatoprotective, vermifuge, and cholagogue [82]. Leaf paste is externally applied for wound healing [39].* S. occidentalis* leaf extracts were found to be active against different microbes [39] and inhibit the growth of 7* M. ulcerans* strains and isolates with MIC mean value of 40 *μ*g/mL (cited as 1 : 5 (20% w/v)) (Table 1) [5]. The presence of compounds such as acrosin, aloe-emodin, emodin, anthraquinones, anthrones, sitosterols, tannins, and xanthosine [39] can justify its reported antimycobacterial activity.

#### 2.2.11. *Psidium guajava* L

*Psidium guajava*, commonly known as guava, is a native plant of tropical America and has been used in indigenous system of medicine for the treatment of various human ailments such as wounds, ulcers, bowels, and cholera [40, 83]. In Central and West Africa, its decoctions are used externally for skin ulcers [84].* P. guajava* also possesses antibacterial and anti-inflammatory properties and also inhibited the growth of 7* M. ulcerans* strains and isolates with MIC mean value of 40 *μ*g/mL (cited as 1 : 5 (20% w/v)) (Table 1) [5]. A number of secondary metabolites in good yield have been isolated and some, which are mainly phenolic, flavonoid, carotenoid, volatile oil, tannins, terpenoid, and triterpene, have been shown to possess useful biological activities [40].

#### 2.2.12. *Capsicum annum* L

*Capsicum annum* is a perennial shrub, with woody trunk, which bears green fruits that ripen to red. The active ingredient in the plant is capsaicin that is used for the management of various medical conditions [48]. This plant possesses antibacterial and wound healing properties. It is used often in combination with* Pothos scandens* L. and* Allium sativum* to heal wounds [85]. The maceration of fruit, used to treat Buruli ulcer, has showed MIC mean value of 40 *μ*g/mL (cited as 1 : 5 (20% w/v)) on 7* M. ulcerans* strains and isolates (Table 1) [5]. Bioactive chemical compounds against bacteria involved in wound infection found in* C. Annum* included saponins, alkaloids, quaternary bases, anthracenosides, flavanosides, flavonds, coumarin derivatives, steroid glycosides, anthocyanosides, essential oils, waxes, coloured materials (mainly capsanthin, capsorubin, zeaxanthin, cryptoxanthin, and lutein), and several capsaicinoids [48, 49].

#### 2.2.13. *Solanum torvum* Sw

*Solanum torvum*, commonly known as Turkey berry, is an erect spiny shrub about 4 m tall, evergreen and widely branched found in Africa and West Indies. The fruits and leaves are widely used in Cameroonian folk medicine. Agrawal et al. [86] have reported the traditional use of this plant as an antiulcer agent while its antimicrobial properties of the leaves are known and are used to manage cuts and wounds [87]. Its leaf decoction inhibits the growth of 7* M. ulcerans* strains and isolate with MIC mean value of 40 *μ*g/mL (cited as 1 : 5 (20% w/v)) (Table 1) [5].* S. torvum* contains a number of potentially pharmacologically active chemicals like stigmasterol involved in the wound healing process and isoflavonoid sulfate and steroidal glycosides, chlorogenone and neochlorogenone, triacontane derivatives, 22-*β*-O-spirostanol oligoglycosides, 26-O-*β*-glucosidase, tetratriacontanoic acid, sitosterol, stigmasterol, campesterol, and C-22 steroidal lactone saponins [50, 86].

#### 2.2.14. *Spathodea campanulata* P. Beauv

*Spathodea campanulata* is a tree that grows between 7 and 25 meters (23–82 feet) tall. It is native to tropical Africa and Southern Asia.* S. campanulata* flowers and bark are used traditionally in the treatment of mental disorders, malaria, hemorrhoids, bacterial infections, HIV, poor blood circulation, gastrointestinal diseases, respiratory ailments, and genital-urinary system disorders [24] as well as relief for skin conditions, swollen cheeks, and body rashes and Buruli ulcer. The leaves and stem bark paste are used to bandage ulcers while infusions of the leaves, root, and bark are also used to clean ulcers [4, 5, 20, 88, 89]. The decoction of the root showed inhibitory activity on 7* M. Ulcerans* strains isolates with MIC mean value of 25 *μ*g/mL (cited as 12.50% (V/V)) (Table 1) [5]. Biologically active phytochemicals have been identified such as alkaloids, tannins, saponins, glycosides, anthraquinone glycosides, steroids, flavonoids, tannins, and glycosides [24–26].

#### 2.2.15. *Allium sativum* L

*Allium sativum* has been widely recognized as a valuable spice and a popular remedy for various ailments and physiological disorders. The plant appears to have originated from Central Asia and then spread to China, the Near East, and the Mediterranean region before moving west to Central and Southern Europe, Northern Africa, and Mexico [90]. The juice of* A. sativum* cloves is used to treat Buruli ulcer conditions [5]. It has been shown experimentally to accelerate the wound healing process in mice [91]. Besides, aqueous extract of* A. sativum* in combination with honey has shown acceleration of wound healing in rats [92]. It has shown growth inhibition on 7* M. ulcerans* strains and isolates with MIC mean values of 0.78–6.25 *μ*g/mL (cited as 0.39–3.13% (V/V)) (Table 1) [5]. Active constituents such as alliin, allicin and *γ*-glutamylcysteine, ajoenes, and vinyldithiins have been identified [9, 10]. Allicin has antimicrobial effects against many bacteria and fungi [90] usually found in ulcers. Its activity on* M. ulcerans* combined with its antibacterial, antifungal and anti-inflammatory, antioxidant, [90, 93], and wound healing potency supports its traditional use in Buruli ulcer treatment.

#### 2.2.16. *Syzygium aromaticum* (L.) Merr. & L. M. Perry

*Syzygium aromaticum* is an evergreen tree found worldwide [41]. It is a natural analgesic and antiseptic used primarily in dentistry because of its main ingredient eugenol. It has been traditionally used externally or locally for the treatment of minor infections of the mouth and skin, dressing of minor wounds, and Buruli ulcer [5]. Its seeds have showed MIC mean value of 25 *μ*g/mL (cited as 12.50% (V/V)) on 7* M. ulcerans* isolates (Table 1) [5]. Many active ingredients have been identified from* S. aromaticum* including essential oils, tannins, gallotannic acid, methyl salicylate, flavonoids eugenin, and triterpenoids like oleanolic acid [41].

#### 2.2.17. *Hydrastis canadensis* L

*Hydrastis canadensis* is an herbaceous perennial growing short yellowish rhizome [94].* H. canadensis* is widely used to treat many ailments, including arrow wounds [46]. It inhibited the growth of 7* M. ulcerans* strains and isolates with MIC values of 0.39–6.25 *μ*g/mL (cited as 0.20–3.13% (V/V)) on (Table 1) [5]. In an antimicrobial screening program,* H. canadensis* extract also exhibited significant activity against multiple drug resistant strains of* M. tuberculosis* and other* Mycobacterium* species as well as other human pathogens [95].* H. canadensis* is found to contain alkaloids, flavonoids, organic acids, sterols (*β*- sitosterol 3-O-*β*-D-glucoside), volatile oil, resin, and fatty acids [46]. Bioassay-guided fractionation revealed berberine to be the active constituent on mycobacteria [95]. This in combination with its wound healing properties can explain the higher activity observed against* M. ulcerans* and support the indigenous treatment of Buruli ulcer.

#### 2.2.18. *Zanthoxylum zanthoxyloides* (Lam.) Zepern. & Timler

*Zanthoxylum zanthoxyloides* is widely distributed in many African countries. It is well known for its use in treating elephantiasis, toothache, sexual impotence, gonorrhoea, malaria, dysmenorrhoea abdominal pain [96], and Buruli ulcer [5]. It showed inhibitory activity with MIC values 12.5–25-*μ*g/mL (cited as 6.25–12.5% (V/V)) on 7* M. ulcerans* strains and isolates (Table 1) [5] in addition to its antimicrobial activity [5, 96]. Antibacterial and anti-inflammatory amides have also been isolated from the plant [96] as well. The presence of a diversity of essential oils, alkaloids, and several aliphatic and aromatic amides [47] strengthens claims of effectiveness of this plant in its traditional use for treatment of Buruli ulcer.

#### 2.2.19. *Gratiola officinalis* L

*Gratiola officinalis* Linn. is a glabrous perennial herb and is native to the south of Europe, and its favourable habitat is damp grounds.* Gratiola officinalis* L., commonly known as common Hedgehyssop or “Herb of Grace” is well known for its pharmacological properties. Various parts of this plant (root and herb) are used in phytomedicines to treat skin diseases [42]. This may explain the observed good inhibitory activity on 7* M. ulcerans *strains and isolates with MIC values 1.56–25-*μ*g/mL (cited as 0.78–12.5% (V/V)) (Table 1) [5]. The active constituents found in* G. officinalis* include gratiogenin, 16-hydroxygratiogenin, cucurbitacins E and I, glycosides gratiogenin-3beta-D-glucoside, gratioside, elaterinide, flavonoids, alkaloids, lignans, coumarin, and saponins which have many biological properties [42].

#### 2.2.20. *Jatropha curcas* L

*Jatropha curcas* Linn belonging to the family Euphorbiaceae is a drought-resistant shrub originating in Central and South America but now thrives in many parts of the tropics and subtropics in Africa and Asia [97].* J. curcas* has been used as traditional medicine to cure Buruli ulcer [4]. Its leaf ethanolic extract inhibited the growth of* M. ulcerans* ATCC 19423 with MIC value of 250 *μ*g/mL and the crude bark extract of* Jatropha curcas* has been shown to be very effective in accelerating wound healing process in rat [98]. Researchers have isolated and characterized a number of biologically active constituents such as flavonoids, apigenin, vitexin, isovitexin, stigmasterol, *β*-D-sitosterol, *β*-D-glucoside, sapogenins, alkaloids, triterpene alcohol, and 1-triacontanols from all parts of this plant [32].

#### 2.2.21. *Holarrhena floribunda* (G. Don) T. Durand

*Holarrhena floribunda* grows as a shrub or tree up to 25 m tall, with a stem diameter of up to 30 cm [4, 21]. Bark is used as an enema or in baths to treat skin infections and the leaf sap is sprinkled on wounds as a haemostatic. A sap extracted from its leaves is sprinkled onto wounds to act as a haemostatic [22]. Yemoa et al. [4] report its use in the traditional treatment of Buruli ulcer and hydroethanolic extract and CH_2_Cl_2_ fraction of root inhibited the growth of* M. ulcerans* ATCC 19423 with MIC value of 125 *μ*g/mL [20] while that of the alkaloid enriched fraction had an MIC of 62.5 *μ*g/mL. These give some support to the use of this plant in traditional medicine (Table 1) [21]. It contains active compounds such as saponins, polar steroidal glycosides, steroidal glycosides, and alkaloids including holaphylline, holaphyllamine, holamine, holaphyllinol, holaphyllidine, holadysamine, holarrhesine, conessine, and progesterone [21, 22].

#### 2.2.22. *Sorindeia juglandifolia* (A. Rich.) Planch. ex Oliv

*Sorindeia juglandifolia* is a shrub or small tree that grows to 23 m tall and 40 cm in diameter. It is widespread in the West and Central Africa subregion. This plant is found on the edges of dry deciduous forest and regrowth in humid forest and in the galleried Sudanian forest of Senegal to Dahomey and also in Ubangi-Shari, Angola, and Zambia. Its common English name is “damson” [99, 100]. In Senegal pulped leaves are applied to sores and ulcers [101]. Fruit fractions and a purified compound (2,3,6-trihydroxymethyl benzoate) from* S. juglandifolia *is reported to have an antimycobacterial activity against* M. ulcerans* strain 1615 with MIC value of 62.5 *μ*g/mL and minimal bactericidal concentration (MBC) values of 250 and 125 *μ*g/mL, respectively [11].

#### 2.2.23. *Annickia chlorantha* (Oliv.) Setten & Maas

*Annickia chlorantha* is a tree up to 30 m tall commonly known as “Yello Wood” found in dense forests in Cameroon, Nigeria, and Gabon. In the southern forest zone of Cameroon, it is used for the traditional treatment of stomach problems, jaundice, urinary tract infections, malaria, tuberculosis, hepatitis, and some forms of ulcer [3, 15]. The stem bark and stem preparations of* A. chlorantha* have been shown to have high inhibition against the growth of* M. ulcerans* strain 1615 with respective MIC values of 1.95 and 7.81 *μ*g/mL (Table 1). The chemistry of* A. chlorantha* formally* Enantia chlorantha* has been extensively studied. Berberine and protoberberine alkaloids [15] with antibacterial [102] properties have been isolated from the stem bark of* A. chloranta*. A mixture of protoberberine alkaloids from* A. chlorantha* containing palmatine, jatrorrhizine, and columbamine was shown to prevent liver injury from chemically induced traumatization and also promoted the healing process after injury in experimental mice [16].

#### 2.2.24. *Greenwayodendron suaveolens* (Engl. & Diels) Verdc

*Greenwayodendron suaveolens* is a deciduous, medium-sized to fairly large tree up to 35 (to 45) m tall that is widespread from Southern Nigeria, East to Western Uganda, Northern Tanzania, and Southern Democratic Republic of Congo and Cabinda (Angola) and commonly known in English as “Molinda.” Various plant parts are used in traditional medicine to treat stomach ache and other pains, gonorrhoea, psychosis, rheumatism, epilepsy and toothache, malaria, liver complaints and headache, helminths, oedema and swollen glands, and hepatitis; it is used to manage infertility, as diuretic, purgative, an aphrodisiac, and to facilitate childbirth [17]. Extracts and fractions from* G. Suaveolens* (cited as* Polyalthia suaveolens*) have been shown to inhibit* M. ulcerans* with moderate MIC and MBC values of 3,125 *μ*g/mL (Table 1) [11]. Many compounds such as polysin, greenwayodendrin-3-one, 3-O-acetyl greenwayodendrin, N-acetylpolyveoline, polyveoline [18], and several alkaloids including indolosesquiterpenes and aporphines have been isolated from the plant [17].

#### 2.2.25. *Phyllanthus amarus* Schumach. & Thonn

*Phyllanthus amarus* is a small herb well known for its medicinal properties and widely used worldwide [44]. It is useful in gastropathy, diarrhoea, dysentery, intermittent fevers, ophthalmopathy, scabies, ulcers, and wounds [44]. The aqueous extract of* P. amarus*, used in traditional medicine in Ivory Coast to treat incurable wounds, has demonstrated* in vitro* activity against* M. ulcerans* strain 02003 with IC_50_ and IC_90_ values, respectively, of 3.5 mg/mL and 19.8 mg/mL and was bactericidal at concentrations of 64 mg/mL [103]. Coulibaly et al. [43] have demonstrated the growth inhibition activity of aqueous and ethanolic extracts of* P. amarus* on 7* M. ulcerans* isolates* in vitro* with similar MIC of 32 mg/mL (Table 1).* P. amarus* have numerous active phytocompounds such as alkaloids, flavonoids, tannins, lignans, polyphenolic compounds, and tetracyclic triterpenoids [44]. Though the main use of* P. amarus* traditionally is to treat tuberculosis [104] rather than Buruli ulcer, its* in vitro* effect on* M. ulcerans* supports its traditional use against Buruli ulcer [43].

#### 2.2.26. *Sacoglottis gabonensis* (Baill.) Urb

*Sacoglottis gabonensis* is a large, evergreen tree up to 40 m tall and it is used to cure many ailment, namely, difficult cases of dermatitis [34] and Buruli ulcer. A decoction of the stem bark of the plant is administered orally or used topically to disinfect the ulcer or the ulcer is covered with fine powder of the stem bark and bandaged [33]. The aqueous extract of* S. gabonensis* showed promising activity against* M. ulcerans in vitro* (MIC = 780 *μ*g/mL) [33] (Table 1). Bergenin, an isocoumarin, was identified as the main active compound of the stem bark extract of* S. gabonensis* [34]. Further analyses of stem bark extract have shown tannins, sterols, polyterpenes, polyphenols, flavonoids, and alkaloids in appreciable amounts with a trace of saponins. The stem bark also contains 2 cis/trans isomers of lignans (calopiptin and galgravin) [34]. Sofowora [35] has also reported that these compounds are responsible for the biological activities of the plant. Kamanzi [105] also argued that plants show antibacterial activity when they contain flavonoids, tannins, saponins, and alkaloids.

#### 2.2.27. Tonic 1: Mixture of* Zea mays* L. and* Spigelia anthelmia* L

Mixture of* S. anthelmia* leaves and* Z. mays* (tonic 1) used in traditional BU treatment [5] showed growth inhibition of 7* M. ulcerans* with MIC values of 6.25–25 *μ*g/mL (cited as 3.13–12.5% (V/V)) (Table 1) [5].

*(i) Spigelia anthelmia L. Spigelia anthelmia* is a common annual weed that grows in open regrowths, on unused land in towns and on road sides [106]. The plant is reputed to be useful in wound healing. A decoction of leaves and twigs is used to wash the wound, which is then dressed with a powder of the bark of the plant [107]. Phytochemical investigation has described the isolation of the alkaloid spiganthine, volatile alkaloids, isoquinoline and actinide isomer, three quaternary alkaloids, choline, benzoylcholine and 2,3-dimethylacrolyl choline, phenylcarboxylic acids, and flavonoids [54, 55] from the plant.

*(ii) Zea mays L. Zea mays* is a robust annual grass up to 4(–6) m tall [108] that possesses wound healing activity [109].* Z. mays* corn silk is rich in phenolic compounds, particularly flavonoids. It also consists of proteins, vitamins, carbohydrates, calcium, potassium, magnesium and sodium salts, volatiles oils, and steroids such as sitosterol and stigmasterol, alkaloids, and saponins [56] that might promote the wound healing and antibacterial activities of the mixture.

#### 2.2.28. Tonic 2: Mixture of* Citrus aurantifolia* (*Christm.*) Swingle and* Gossypium barbadense* L

It consists of* C. aurantifolia* mixed with* G. barbadense* and inhibits the growth of 7* M. ulcerans* isolates with MIC values of 12.5–25 *μ*g/mL (cited as 6.25–12.5% (V/V)) (Table 1) [5].

*(i) Citrus aurantifolia (Christm.) *Swingle.* Citrus aurantifolia* is widespread in tropical and subtropical regions around the world and it is known for its nutritional values and flavour [110]. The plant and fruit of* C. aurantifolia* have been commonly used in traditional medicine to treat various diseases [110] including Buruli ulcer. It is used either alone as decoction or mixed with* G. barbadense* leaves or* S. campanulata* as a bandage for ulcer [4, 5, 20, 68, 88]. The essential oil of* C. aurantifolia* fruits contain mainly limonene, beta-pinene, gamma-terpinene, and citral [111]. The methanolic extract of* C. aurantifolia* contains alkaloids, flavonoids, tannins, saponins, steroids, cardiac glycosides, and reducing sugar [57]. The hexane extract of the fruit peels fractionated by column chromatography yielded the following major compounds: 5-geranyloxypsoralen, 5-geranyloxy-7-methoxycoumarin, 5,7-dimethoxycoumarin, 5-methoxypsoralen, and 5,8-dimethoxypsoralen. In addition, GC-MS analysis of the hexane extract allowed for the identification of 44 volatile compounds, where 5,7-dimethoxycoumarin, 3-methyl-1,2-cyclopentanedione, 1-methoxy-ciclohexene, corylone, palmitic acid, 5,8-dimethoxypsoralen, *α*-terpineol, and umbelliferone are the major constituents. Some constituents that have shown activity against* Mycobacterium tuberculosis* strains were 5,8-dimethoxypsoralen (MICs = 25–50 *μ*g/mL), 5-geranyloxypsoralen (MICs = 50–100 *μ*g/mL), palmitic acid (MICs = 25–50 *μ*g/mL), linoleic acid (MICs = 50–100 *μ*g/mL), oleic acid (MICs = 100 *μ*/mL), 4-hexen-3-one (MICs = 50–100 *μ*g/mL), and citral (MICs = 50–100 *μ*g/mL) [110]. The antimycobacterial activity of* C. aurantifolia* against* M. ulcerans* could be attributed to these compounds and might have significantly contributed to the observed potency of the mixture.

*(ii) Gossypium barbadense L. G. barbadense* is an annual herb that has been reported to have many therapeutic effects including treatment of cutaneous and subcutaneous parasitic infections are mostly attributed to its active constituent gossypol [58].

#### 2.2.29. Tonic 3: Mixture of* Jatropha curcas*,* Gossypium hirsutum*,* Physalis angulate*, and* Delonix regia*

Tonic 3 consisting of* J. curcas* mixed with* G. hirsutum*,* P. angulata*, and* D. regia* inhibits the growth of 7* M. ulcerans* isolates with MIC value of 6.25–25 *μ*g/mL (reported as 3.13–12.5% (V/V)) (Table 1) [5].

*(i) Jatropha curcas L.* See the above.

*(ii) Physalis angulata L. Physalis angulata* is a much branched annual shrub, perennial in subtropical zones, and can grow until it reaches 1.0 m. It is used in several countries of tropical and subtropical regions of the world as medicinal and fruit tree. Recent ethnopharmacological studies show that* P. angulata* leaf paste is used as an external application for wounds [59]. The major identifiable phytochemical constituents of medicinal importance are physalins and withanolides [59, 60]. The anti-inflammatory, antimycobacterial, antinociceptive, and antitumor activities together with the inhibitory effect on* M. Ulcerans* support its traditional uses for Buruli ulcer treatment.

*(iii) Gossypium hirsutum L. Gossypium hirsutum* is a perennial shrubs also known as upland cotton or Mexican cotton. It is native to Mexico. Extract from cotton plant, which would be primarily gossypol, has been used as traditional medicine. Cotton leaves have been used as a treatment for nausea during pregnancy, for “proud flesh” (swollen tissue around a wound) or for fungal infections. Cotton tissue, particularly the seeds, can be toxic if ingested in excessive quantities because of the presence of antinutritional and toxic factors including gossypol and cyclopropenoid fatty acids (including dihydrosterculic, sterculic, and malvalic acids) [61, 62].

*(iv) Delonix regia (Hook.) *Raf.* Delonix regia* is broad, spreading, flat crowned deciduous tree found in tropical areas. Phytochemical studies of* D. Regia* have shown the presence of sterols, triterpenoids, phenolic compounds, flavonoids, sugars, tannins, steroids, *β*-sitosterol, lupeol, hydrocarbons phytotoxins, saponins [63, 64], and an aromatic compound, p-methoxybenzaldehyde. Adje et al. [65] characterized three major anthocyanins: cyanidin 3-O-glucoside, cyanidin 3-O-rutinoside, and pelargonidin 3-O-rutinoside; three sterols (stigmasterol, *β*-sitosterol, and its 3-O-gucoside); a triterpene (ursolic acid) and four flavonoids (quercetin, quercitrin, isoquercitrin, and rutin) [112]. Ethanolic and aqueous extracts of* D. regia* flowers containing *β*-sitosterol and stigmasterol significantly promoted the healing process in rat [113] supporting its usage in the treatment of Buruli ulcer.

### 2.3. Anti-*Mycobacterium ulcerans* Compounds Isolated from Plants

**Figure 1:**
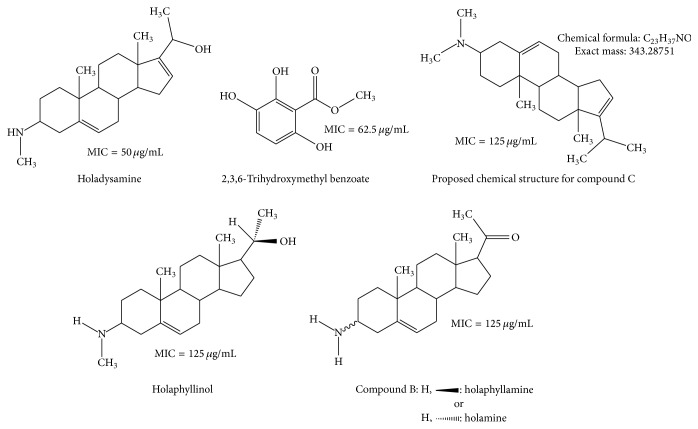
Potent anti-*Mycobacterium ulcerans* compounds isolated from plants.

Five active compounds with MICs ranging from 50 to 125 *μ*g/mL have been isolated from two plants: one from* Sorindeia juglandifolia* (2,3,6-trihydroxymethyl benzoate) [11] and four from* Holarrhena floribunda* (holadysamine, holaphyllinol, holamine/holaphyllamine, compound C required further analysis to confirm the structure) [21] (see Figure 1).

## 3. Conclusion and Future Perspectives

In this review, we have discussed medicinally significant plant species from Sub-Saharan Africa and showed that many have activity against* M. ulcerans*. Currently, there are only two studies that have reported the purification of active compounds against* M. ulcerans*. This highlights the poor emphasis given to research into new chemotherapeutic agents against one of the world most neglected diseases:* Mycobacterium ulcerans* disease. The present review can be used to validate ethnomedicinal knowledge and bioactivities. Unfortunately, most of the species that are claimed to contain antimycobacterial activities have not been studied* in vivo*. Screening with* in vitro* assays has little meaning if there is no clear evidence of effectiveness of the extracts* in vivo*. Therefore, further* in vivo* studies of preparations from the identified plant species are required. This should be followed by systematic phytochemical studies of plants that contain bioactive antimycobacterial activities, isolation, and characterization of the anti-*M. ulcerans* chemical entities. These can provide the needed validation before such chemical entities can be used as sustainable cheaper/alternative medicines for management of Buruli ulcer disease.

## Figures and Tables

**Table 1:** Extraction methods, main components, and antimycobacterial potential of medicinal plant species.

Family	Species	Part used	Extraction method	Solvent(s) used	Main components (or groups)	Antimycobacterial assay methods	Activity/MIC (*µ*g/mL)	Reference
Amaranthaceae	*Pupalia lappacea *Juss.	Leaves	Hot water extraction	Water	Alkaloids, amino acids, glycosides, flavonoids, glycosides, saponins, tannins, starch, coumarins, terpenoids, and steroids such as 1-docosanol, stearic acid, stigmasterol, sitosterol, N-benzoyl-L-phenylalaninol acetate, setosterol-3-O-D-glucopyranoside, stigmasterol-3-O-D-glucopyranoside, and 20-hydroxyl ecdysone	Proportion method	40 (cited as 1 : 5 (20% w/v))	[5–8]

Amaryllidaceae	*Allium sativum *L.	Bulb large/white cloves	Cold maceration (juice)	Water	Alliin, *γ*-glutamylcysteine peptides, allicin, ajoenes, vinyldithiins, and sulfides	Proportion method	0.78–6.25 (cited as 0.39–3.13% (V/V))	[5, 9, 10]
		Bulb small/purple cloves	Water/juice				3.13–6.25 (cited as 1.56–3.13% (V/V))	[5]

Anacardiaceae	*Sorindeia juglandifolia* A. Rich.	Fruit	Cold maceration, column chromatographies (Fractions SJfr 3.2 and SJfr 3.41)	Methanol, hexane, ethyl acetate, chloroform	C-glycosylflavone, 2′′,6′′-di-O-acetyl-7-O-methyl vitexin, 2′′-O-acetyl-7-O-methyl vitexin, mearnsitrin, robustaflavone, 3-O-galloyl catechin, tachioside, 3о-O-D-glucopyranosyl-о-stigmasterol, methyl gallate, 2,3,6-trihydroxybenzoic acid, 2,3,6-trihydroxymethyl benzoate	REMA	62.5	[11–13]
	*Lannea nigritana *(Sc. Elliot) Keay	Root	Decoction	Water	Tannins and phenolic compounds (Lanneanol)	Proportion method	40 (cited as 1 : 5 (20% w/v))	[5, 14]

Annonaceae	*Annickia chloranta *(Oliv.) Setten and Maas	Stem bark	Maceration	Ethanol	Berberine and protoberberine alkaloids: palmatine, jatrorrhizine, columbamine	REMA	1.95	[11, 15, 16]
		Stem	Interface water-CH_2_Cl_2_/ACstI	Partitioned between water and dichloromethane			7.81	
	*Greenwayodendron suaveolens* (Engl. & Diels) Verdc. (cited as *Polyalthia suaveolens* Engl. & Diels)	Stem bark	Maceration	Ethanol, partitioned between water and dichloromethane and methanol and hexane (methanol fraction)	Polysin, greenwayodendrin-3-one, 3-O-acetyl greenwayodendrin, N-acetylpolyveoline, polyveoline, indolosesquiterpenes, aporphines	REMA	3125	[11, 17, 18]

Apocynaceae	*Alstonia boonei *De Wild.	Leaves	Hot water maceration	Water	Alkaloids (echitamine, echitamidine, voacangine, akuammidine, N-formylechitamidine, N*α*-formyl-12-methoxyechitamidine), tannins, iridoids (boonein, loganin), triterpenoids (lupeol, ursolic acid, *β*-amyrin)	Proportion method	40 (cited as 1 : 5 (20% w/v))	[5, 19]
	*Holarrhena floribunda *(G. Don) T. Durand et Schinz	Root	Maceration	70% ethanol	Saponins, polar steroidal glycosides, steroidal glycosides, alkaloids including holaphylline, holaphyllamine, holamine, holaphyllinol, holaphyllidine, holadysamine, holarrhesine, conessine, conanine, conamine, progesterone, norconessine (kurchine), conessimine, kurchamine, conimine, conamine, holarrhenine, holarrhimine, conarrhimine	REMA	125	[20–22]
			Liquid chromatography	Hexane, dichloromethane, ethyl acetate, and water (CH_2_Cl_2_ fractions)			125	[20]
			Fractionation	70% ethanol, 2% sulfuric acid, hexane, 10% NaOH, dichloromethane (alkaloid enriched fraction extract)			62.5	

Araceae	*Aglaonema commutatum *Schott	Leaves	Hot water maceration	Water	Calcium oxalate crystals	Proportion method	40 (cited as 1 : 5 (20% w/v))	[5, 23]

Bignoniaceae	*Spathodea campanulata P. Beauv.*	Root	Decoction	Water	Carbohydrates, alkaloids, Tannin glycosides, steroids, saponins, anthraquinone glycosides, flavonoids, tannins,glycosides	Proportion method	25 (cited as 12.50% (V/V))	[5, 24–26]

Cleomaceae	*Cleome viscosa *L.	Leaves	Hot water maceration	Water	Alkaloid, tannins, saponins, flavonoids, terpenes, carbohydrates	Proportion method	40 (cited as 1 : 5 (20% w/v))	[5, 27, 28]

Compositae	*Ageratum conyzoides *(L.) L.	Leaves	Hot water maceration	Water	Pyrrolizidine alkaloids lycopsamine, dihydrolycopsamine, acetyl-lycopsamine, *N*-oxides, methoxylated flavonoids, chromenes	Proportion method	40 (cited as 1 : 5 (20% w/v))	[5, 29]

Euphorbiaceae	*Bridelia ferruginea *Benth.	Stem bark	Decoction	Water	Polyphenols, steroids, saponins, tannins, terpenoids, alkaloids, quercetin derivatives such as rutin, myricetin derivatives gallocatechin-(4′-O-7)-epigallocatechin; 3,5-dicaffeoylquinic acid and 1,3,4,5-tetracaffeoylquinic acid; lignans deoxypodophyllotoxin, *β*-peltatin, *β*-peltatin-5-O-*β*-D-glucopyranoside, 5′-demethoxy-*β*-peltatin-5-O-*β*-D-glucopyranoside	Proportion method	40 (cited as 1 : 5 (20% w/v))	[5, 30, 31]
	*Jatropha curcas *L.	Leaves	Maceration	70% ethanol	Flavonoids, apigenin, vitexin, isovitexin, sterol stigmasterol, *β*-D-sitosterol, *β*-D-glucoside, sapogenins, alkaloids, triterpene alcohol, 1-triacontanol	REMA	250	[21, 32]

Humiriaceae	*Sacoglottis gabonensis *(Baill.) Urb.	Stem bark	Cold maceration	Water	Bergenin, sterols, polyterpenes, polyphenols, flavonoids, tannins, saponins, alkaloids	Proportion method	Growth inhibition *in vitro*	[33–38]

Leguminosae	*Senna occidentalis *(L.) Link (Syn.* Cassia occidentalis *L.)	Leaves	Hot water maceration	Water	Acrosin, aloe-emodin, emodin, anthraquinones, anthrones, apigenin, aurantiobtusin, campesterol, cassiollin, chrysoobtusin, chrysophanic acid, chrysarobin, chrysophanol, chrysoeriol, emodin, essential oils, funiculosin, galactopyranosyl, helminthosporine, islandicine, kaempferol, lignoceric acid, linoleic acid, linolenic acid, mannitol, mannopyranosyl, matteucinol, obtusifolin, obtusin, oleic acid, physcion, quercetin, rhamnosides, rhein, rubrofusarin, sitosterols, tannins, xanthosine	Proportion method	40 (cited as 1 : 5 (20% w/v))	[5, 39]

Myrtaceae	*Psidium guajava *L.	Leaves	Hot water maceration	Water	Phenol, tannin (Amritoside: ellagic acid 4-gentiobioside, Guavins A, B, C, and D, isostrictinin (V), strictinin, pedunculagin, (+)-gallocatechin), flavonoid (quercetin and its glycosides, morin-3-O-*α*-L-lyxopyranoside, morin-3-O-*α*-L-arabinopyranoside, kaempferol, luteolin-7-O-glucoside, apigenin-7-O-glucoside), carotenoid, terpenoid, triterpene, isoprenoids (monoterpenes: caryophyllene oxide, *β*-selinene, 1,8-cineole, *α*-pinene, myrcene, *δ*-elemene, d-limonene, caryophyllene, linalool, eugenol, *β*-bisabolol, *β*-bisabolene, *β*-sesquiphellandrene, Me 2-methyl-thiazolidine-4-(R)-carboxylate (cis and trans), ethyl 2-methyl-thiazolidine-4-(R)-carboxylate (cis and trans), aromadendrene, *α*- and *β*-selinene, caryophyllene epoxide, carophylladienol, (E)-nerolidol, Selin-11-en-4-alpha-ol, and terpenoids: guavanoic acid, guavacoumaric acid, guajanoic acid, ursolic acid, 2*α*-hydroxyursolic acid, Maslinic acid, asiatic acid, Jacoumaric acid, isoneriucoumaric acid, guajavanoic acid, guajavolide, guavenoic acid)	Proportion method	40 (cited as 1 : 5 (20% w/v))	[5, 40]
	*Syzygium aromaticum *(L.) Merr. & L.M. Perry	Seed	Decoction	Water	Essential oils, eugenol, eugenyl acetate, *β*-caryophyllene, methyl amyl ketone, *α*- and *β*-humulene, benzaldehyde, methyl salicylate, *α*-cubebene, *α*-copaene, *γ*-cadinene and *δ*-cadinene *β*-ylangene and chavicol vanillin, crategolic acid, tannins, gallotannic acid, flavonoids eugenin, kaempferol, rhamnetin, eugenitin, triterpenoids like oleanolic acid	Proportion method	25 (cited as 12.50% (V/V))	[5, 41]

Plantaginaceae	*Gratiola officinalis *L.	Bark	Decoction	Water	Alkaloid, flavonoid, saponins, coumarin derivatives, mannitol, glycoside-like substances, Gratiogenin, 16-hydroxygratiogenin, cucurbitacins E and I, glycosides gratiogenin-3beta-D-glucoside, gratioside, elaterinide, lignans	Proportion method	1.56–25 (cited as 0.78–12.5% (V/V))	[5, 42]

Phyllanthaceae	*Phyllanthus amarus *Schumach. & Thonn.	Leaves	Maceration	70% ethanol	Alkaloids, flavonoids, tannins (geraniin, corilagin, 1,6-digalloylglucopyranoside rutin, quercetin-3-O-glucopyranoside, amarulone, phyllanthusiin D and amariin), lignans (niranthin, nirtetralin, phyltetralin, hypophyllanthin, phyllanthin, demethylenedioxy-niranthin, 5-demethoxy-niranthin, isolintetralin), polyphenolic compounds, tetracyclic triterpenoids	Proportion method	32000	[43, 44]
	*Phyllanthus fraternus *G.L. Webster	Leaves	Hot water maceration	Water	Alkaloids (phyllanthin, hypophyllanthin, nirphyllin, phyllnirurin, phyllanthol, phyllanthenol, rhamnopyranoside, phyllanthenone, lintetralin, astragalin, cymene, niranthin, nirtetralin, niruriside, phyllochrysine, 4-methoxy-securinine, 4-methoxy-nirsecurinine, limonene, niruretin, nirurin, phyllochrysine), steroids (*β*-sitosterol, cholesterol), flavonoids (quercetin, quercetin heteroside, quercetol, quercitrin, 3, 4, 5-trimethoxy flavanone, 3,5,7-trihydroxy flavonol), other compounds (estradiol, corilagin, ellagic acid, gallic acid, rutin, gernanine, rutinoside, lupa, lupeol, methyl salicylate), saponins (triacontanal, triacontanol)	Proportion method	40 (cited as 1 : 5 (20% w/v))	[5, 45]

Ranunculaceae	*Hydrastis canadensis *L.	Root	Decoction	Water	Alkaloids (major: hydrastine, berberine, canadine; minor: hydrastinine, canadaline, isohydrastidine, 1-*β*-hydrastine, 5-hydroxytetrahydroberberine, (S)-corypalmine, (S)-isocorypalmine, (S)-tetrahydropalmatine, Berberastine, 8-oxotetrahydrothalifendine, Canadinic acid), flavonoids (6,8-C-dimethylluteolin 7-methyl ether, 6-C-methylluteolin 7-methyl ether, sideroxylin, 8-desmethyl-sideroxylen, 6-desmethyl-sideroxylin), organic acids (quinic acid derivatives, hycandinic acid esters, 5-O-(4′-[*β*-d-glucopyranosyl]-trans-feruloyl) quinic), Sterols (*β*-sitosterol 3-O-*β*-D-glucoside), volatile oil, resin, and fatty acids	Proportion method	0.39–6.25 (cited as 0.20–3.13% (V/V))	[5, 46]

Rutaceae	*Zanthoxylum zanthoxyloides *(Lam.) Zepern. & Timler	Roots	Decoction	Water	Essential oils, benzophenanthridine, furoquinoline, aporphine alkaloids, fagaronine, dihydroavicine, chelerythrine, oxychelerythrine, skimmianine and 8-methoxydictamine, as well as the aporphines magnoflorine, berberine, tembetarine, N-methyl-corydine, N-isobutyldeca-2, 4-dienamide, N-isobutylocta-2,4-dienamide, arnottianamide, fagaramide, piperlonguminine, rubemamin, N-isopentyl-cinnamamide, umbelliferone, scopoletin, scoparone, xanthotoxin, imperatorin, bergapten, marmesin, pimpinellin; lignan sesamin, C-7 epimer asarinin, sterols zanthoxylol, diosmin, fagarol, hesperidin, lupeol, *β*-sitosterol, stigmasterol, campesterol, *β*-amyrin,: vanillic acid, hydroxybenzoic acid, parahydroxybenzoic acid, 2-hydroxymethyl benzoic acid, parafluorobenzoic acid, divanilloylquinic acids burkinabin A, burkinabin B, and burkinabin C	Proportion method	12.5–25 (cited as 6.25–12.5% (V/V))	[5, 47]

Solanaceae	*Capsicum annum *L.	Fruit	Cold maceration	Water	Saponins, alkaloids, quarternary bases, anthracenosides, flavanosides, flavonds, coumarin derivatives, steroid glycosides, anthocyanosides, essential oils, waxes, capsanthin, capsorubin, zeaxanthin, cryptoxanthin, lutein, capsaicinoids	Proportion method	40 (cited as 1 : 5 (20% w/v))	[5, 48, 49]
	*Solanum torvum *Sw.	Leaves	Hot water maceration	Water	Isoflavonoid sulfate, steroidal glycosides, chlorogenone, neochlorogenone, triacontane derivatives, 22-*β*-O-spirostanol oligoglycosides, 26-O-*β*-glucosidase	Proportion method	40 (cited as 1 : 5 (20% w/v))	[5, 50]

Xanthorrhoeaceae	*Aloe vera *(L.) Burm. f.	Leaves	Cold maceration	Water	Chromone, anthraquinone, anthrone derivatives, anthraquinones, saponins, salicylic acids, amino acids, vitamins, enzymes, minerals, sugars, lignin	Proportion method	40 (cited as 1 : 5 (20% w/v))	[5, 51, 52]

Tonic 1	Mixture of* Spigelia anthelmia *L. and* Zea mays *L.	Leaves, grain	Hot water maceration	Water	*Spigelia anthelmia*: alkaloid spiganthine, ryanodine, volatile alkaloids, isoquinoline, actinide isomer, quaternary alkaloids, choline, benzoylcholine, 2,3-dimethylacrolyl choline, phenylcarboxylic acids, flavonoids *Zea mays*: proteins, vitamins, carbohydrates, volatiles oils, steroids such as sitosterol and stigmasterol, alkaloids, saponins	Proportion method	6.25–25 (cited as 3.13–12.5% (V/V))	[5, 53–56]

Tonic 2	Mixture of *Citrus aurantifolia *(Christm.) Swingle and* Gossypium barbadense *L.	Leaves	Hot water maceration	Water	*C. aurantifolia*: alkaloids, flavonoids, tannins, saponins, steroids, cardiac glycosides, reducing sugar, 5-geranyloxypsoralen, 5-geranyloxy-7-methoxycoumarin, 5,7-dimethoxycoumarin, 5-methoxypsoralen, 5,8-dimethoxypsoralen, 3-methyl-1,2-cyclopentanedione, 1-methoxy-ciclohexene, corylone, palmitic acid *α*-terpineol, umbelliferone *Gossypium barbadense*: gossypol, 1,1-dimethybutanol, (3-ethyl-2 oxiranyl) ethanone, (3-ethyl-2 oxiranyl) ethanone, 4,11,11-trimethyl-8-methylene bicycle (7.2.0) undec-4-ene, 9-octadecene, hexadecene, 1-(1,5-dimethyl-4-hexany)-4-methyl-3-cyclohexen-1-ol, 9-eicosene, hexadecanoic acid, methyl ester palmitic acid, 3-eicosene, 9,12,15-octadecatrienoic acid, methyl ester linolenic acid, phytol-2-hexadecen-1-ol, oleic acid 9-octadecanoic acid, 1-tricosene, hexadecanoic acid, pentafluoropropionic acid heptadecyl ester, 13-octadecenal, di-n-octylphthalate 1,2-benzenedicarboxylic acid, squalene 2,6,10,15,19,23-hexamethyl	Proportion method	12.5–25 (cited as 6.25–12.5% (V/V))	[5, 57, 58]

Tonic 3	Mixture of *Jatropha curcas *L.*, Gossypium hirsutum *L.*, Physalis angulata L., *and* Delonix regia *(Hook.) Raf.	Leaves	Hot water maceration	Water	*Jatropha curcas*: flavonoids, apigenin, vitexin, isovitexin, sterol stigmasterol, *β*-D-sitosterol, *β*-D-glucoside, sapogenins, alkaloids, triterpene alcohol, 1-triacontanol *Gossypium hirsutum*: gossypol and cyclopropenoid fatty acids including dihydrosterculic, sterculic, and malvalic acids *Physalis angulate*: flavonoids (A flavonol glycoside, myricetin 3-O-neohesperidoside), alkaloids (phygrine) and many different types of plant steroids (physalins) A-W, withanolides, physagulins A, B, C, and D, withanolides, withangulatins B, C, D, E, F, G, H, and I, withangulatin I, physangulidines A, B, and C, carotenoids, and oleanolic acid *Delonix regia*: sterols, triterpenoids, phenolic compounds, saponins, alkaloids, flavonoids, sugars, tannins, steroids, *β*-sitosterol, carotene, hydrocarbons phytotoxins, carotenoids, lupeol, epilupeol, stigmasterol, p-methoxybenzaldehyde, kaempferol 3-rhamnoside 1, quercetin 3-rhamnoside 2, kaempferol 3-glucoide 3, kaempferol 3-rutinoside 4, kaempferol 3-neohesperidoside 5, quercetin 3-rutinoside 6 and quercetin 3-glucoside 7, anthocyanins, cyanidin 3-o-glucoside, cyanidin 3-o-rutinoside, pelargonidin 3-o-rutinoside	Proportion method	6.25–25 (cited as 3.13–12.5% (V/V))	[5, 32, 59–65]

w/v = 20% w/v (200 *µ*g/mL) of herbal preparations (infusions, decoctions and juices) were each incorporated at 1 : 5 dilution into L-J medium corresponding to a final concentration of 40 *µ*g/mL. V/V = The herbal preparations (infusions, decoctions and juices) at 20% w/v were incorporated into L-J media at final concentrations ranging from 25% vol/vol to 0.20% vol/vol corresponding to 50 *µ*g/mL to 0.4 *µ*g/mL.
